# First evidence of hepatitis E virus in domestic pigs: a cross-sectional seroepidemiological study in the Bosnia and Herzegovina

**DOI:** 10.3389/fvets.2025.1667254

**Published:** 2025-10-16

**Authors:** Abdullah Muftić

**Affiliations:** Department of Pathobiology and Epidemiology, Veterinary Faculty, University of Sarajevo, Sarajevo, Bosnia and Herzegovina

**Keywords:** HEV, epidemiology, risk factors, biosecurity, swine

## Abstract

**Introduction:**

Hepatitis E virus (HEV) is foodborne zoonotic pathogen widespread among European swine yet unstudied in Bosnia and Herzegovina (B&H). We estimated HEV seroprevalence in domestic pigs in Federation of B&H (FB&H) and assessed farm-level risk factors for exposure.

**Methods:**

Cross-sectional survey sampled 437 pigs from 87 farms across seven cantons via two-stage random design. Serum anti-HEV IgG measured by commercial indirect ELISA; managers completed standardized biosecurity/management questionnaire. Apparent seroprevalence calculated with 95% CIs. Univariable screening (*α* = 0.10) informed multivariable logistic regression with farm-level clustering; collinearity checked (Phi), AIC-guided forward selection applied.

**Results:**

Animal-level seroprevalence 77.1% (95% CI 73.0–81.0%); herd-level 95.4% (88.9–98.7%). Adults showed higher seropositivity than growers (91.0% vs. 71.7%; *p* < 0.001). Significant factors: wild-boar proximity (adjusted POR 3.11; *p* = 0.04), small farm size (18.35; *p* < 0.001), swill feeding (5.70; *p* = 0.03). Cleaning ≥5×/month strongly protective (0.01; *p* < 0.001). All surveyed cantons had positives; no equivocal ELISA results.

**Discussion:**

Findings indicate widespread HEV in FB&H swine with environmental, food-safety, and occupational implications. Older-animal pattern reflects cumulative exposure; small-farm context and wildlife interface likely sustain transmission, whereas frequent cleaning reduces risk. Strengthened biosecurity, wildlife exclusion, feed oversight (including prohibition/monitoring of swill feeding), and improved hygiene, should form basis of One Health interventions to mitigate potential zoonotic transmission via the pork production chain.

## Introduction

*Paslahepevirus balayani*, more commonly known as Hepatitis E virus (HEV), is a non-enveloped RNA virus that has become recognized as an emerging zoonotic pathogen in recent decades (1–3). Distinct epidemiological patterns of HEV are observed: genotypes 1 and 2 spread via contaminated water in developing regions, whereas genotypes 3 and 4 are zoonotic and cause sporadic, foodborne infections in industrialized countries. In humans HEV can cause acute hepatitis E with outcomes ranging from mild to fulminant. While most infections are self-limiting, severe disease or chronic HEV infection occurs in high-risk groups (e.g., immunocompromised patients), while pregnant women infected with HEV1 may experience case fatality rates as high as 25–30% (4, 5). Importantly, HEV genotype 3 (HEV 3) (predominant in Europe) is highly prevalent in swine population*s* yet causes no apparent illness in pigs, enabling silent on-farm circulation (1). Thus, domestic pigs and wild boars are now recognized as the main reservoir for human-infecting HEV strains in industrialized settings (2, 6). The growing number of autochthonous HEV cases linked to pork consumption in Europe underscores the significance of HEV as an emerging zoonosis at the human–animal interface. Transmission to humans is primarily foodborne, through the consumption of undercooked pork meat or offal (especially liver), or via direct contact with infected pigs (4, 6). Consequently, hepatitis E has transitioned from a travel-associated illness to a public health issue within Europe, driven by the swine reservoir and food chain exposure (7).

HEV infection in European pig herds is endemic and widespread. Surveys across numerous countries consistently reveal high exposure rates, though prevalence estimates vary widely by region and study (2). Most pig farms in Europe (and globally) have evidence of HEV circulation, with farm-level seropositivity often nearing 100%. Within any given farm, however, the infection dynamics can differ markedly—seroprevalence and viral shedding proportions fluctuate with factors such as herd immunity and management practices (6). In Europe, genotype 3 HEV is enzootic in pigs, and the majority of pigs develop anti-HEV antibodies (IgG) by adulthood. For example, recent studies in Italy found almost 90% of slaughter-age swine seropositive (4). Similarly, high HEV seroprevalence in pigs has been documented across the Balkan region in the last decade. A One Health review reported anti-HEV IgG in approximately 20–55% of tested domestic pigs in Serbia, 29–50% in Bulgaria, 39–50% in Romania, and 31–92% in Croatia (8). Country-specific investigations corroborate these findings: in Serbia, around 34–41% of pigs have been seropositive on both smallholder and intensive farms (9), while in Bulgaria seroprevalence estimates range from roughly 40% in initial surveys up to 60% in certain regions (10). Notably, HEV exposure has also been confirmed in Balkan wild boar populations (8, 11), raising concern that wildlife could introduce or disseminate the virus between pig farms. Despite the increasing body of regional evidence, Bosnia and Herzegovina (B&H) has to date no published data on HEV in either humans or domestic swine, constituting a critical knowledge gap. B&H was absent from the recent regional assessments of HEV prevalence (8), highlighting the need for baseline research.

Closing this gap is vital for both veterinary and public health in B&H, as HEV may circulate silently in pigs and present a zoonotic risk. Because infection in swine is typically asymptomatic, effective surveillance must rely on serological and molecular testing. Attention to farm management and biosecurity is essential, since herd size, hygienic practices, and housing conditions can significantly influence transmission dynamics (2, 6, 8). Considering these aspects, this study provides the first serological evidence of HEV in pigs in FB&H, along with its associated risk factors, with the aim of supporting future surveillance, research, and interventions to safeguard both animal and public health in the country.

## Methods

### Study design and sampling

This cross-sectional study classified pigs according to their HEV serostatus in a representative sample of herds, providing both prevalence estimates and an assessment of risk factors. The required sample size was calculated using official swine population figures for the Federation of Bosnia and Herzegovina (FB&H), as reported in the *Green Report* of the Federal Ministry of Agriculture, Water Management and Forestry (12). According to the 2023 report, FB&H contained 88,585 pigs distributed across 5,870 farms in 10 cantons. Pig farms were categorized by herd size following the FAO’s classification system (13). For this study, herd size thresholds were defined as small (<10 pigs), medium (10–29 pigs), and large (≥30 pigs). On average, registered farms in FB&H maintained 4.2 pigs, with most production conducted on a seasonal basis for meat. Pasture-based herding systems predominated among small- and medium-scale farms, whereas batch production systems were mainly characteristic of large-scale operations (12). Cantons contributing less than 0.5% of the national herd were excluded to maintain representativeness. At the animal level, pigs under 3 months of age or deemed unfit for ethical reasons were omitted to avoid false serological results (due to maternal antibodies) and minimize animal distress.

A two-stage random sampling design, adapted from Pickles et al. (14), was employed. In the first stage, farms (clusters) were randomly selected with probability proportional to each canton’s share of the total pig population. In the second stage, pigs were randomly chosen within selected farms. In the absence of prior HEV data for B&H, a conservative estimate of 50% seroprevalence at both farm and animal levels was applied to maximize sample size. Using a 5% margin of error and 95% confidence level, the target sample was estimated at 87 farms and 437 pigs. Randomization at both stages was performed using a computer-based random number generator (Microsoft Excel). The sampling protocol was consistent with that used in our earlier swine toxoplasmosis survey (15).

Blood samples were collected by venipuncture from the selected pigs, following standard veterinary procedures. Serum was separated and stored at −20 °C until testing. All animal handling was performed by trained veterinarians, in line with animal welfare guidelines, and informed consent was obtained from all farm owners. Sampling took place during November and December 2024.

### Serological testing

Sera were tested for HEV-specific antibodies using a commercial indirect ELISA kit (PrioCHECK® HEV Ab porcine, Prionics AG, Schlieren, Switzerland), strictly following the manufacturer’s instructions. Briefly, sera were diluted 1:100 and added to HEV antigen–coated microplate wells. After incubation and washing, a peroxidase-conjugated anti-swine IgG was applied as the detection antibody. Following substrate reaction, absorbance was measured at 450 nm (reference 620 nm) using a BioTek Epoch microplate reader. Each assay run included kit-provided positive and negative controls, along with a calibrator (cut-off control). The cutoff value was calculated as the mean OD450 of the cut-off control wells × 1.2, as specified by the manufacturer. Samples with OD450 ≥ cutoff were classified as seropositive, and those below as negative. The ELISA has a reported diagnostic sensitivity of 91% and specificity of 94.1%.

### Questionnaire and risk factor analysis

Data on potential risk factors for HEV infection were gathered through a standardized questionnaire administered via interview with each farm manager. The selection of candidate variables was informed by a literature review (2, 6, 8), which highlighted factors most frequently cited as determinants of swine HEV infection risk. The questionnaire addressed farm-level sanitation and biosecurity measures such as pen cleanliness and disinfection routines, herd management practices including herd size and production type, the presence of or contact with other animal species—particularly wild boar or other livestock—animal introduction practices such as importation and quarantine, and any history of reproductive or hepatic disorders on the farm. Information on disinfection routines, presence of wild animals, introduction of new pigs, quarantine practices, and history of disorders was obtained through farmer self-reports, whereas all other data were verified directly on-site during the farm visits. These factors have previously been noted as relevant to HEV transmission (16, 17). All variables were recorded as binary or categorical responses as appropriate.

For analysis, apparent seroprevalence was first calculated at both the farm level, defined as the proportion of farms with at least one seropositive pig, and the animal level, defined as the proportion of individual pigs seropositive, with Wilson 95% confidence intervals (CIs). Risk factor evaluation was conducted at the individual pig level, with statistical methods accounting for clustering by farm. Each candidate variable from the questionnaire was initially assessed using univariable analysis. For binary factors, prevalence odds ratios (PORs) with 95% CIs were computed by comparing the category of interest against the reference category. Statistical significance was evaluated with Chi-square tests, or Fisher’s exact test when cell counts were small. To avoid premature exclusion of potentially meaningful predictors, a liberal screening threshold of *α* = 0.10 was applied.

Collinearity among candidate variables was assessed through pairwise associations between binary predictors using the Phi coefficient. When strong correlations were observed (Phi > 0.3), only one of the correlated variables was retained—generally the one with the higher univariable POR or greater biological relevance—to minimize the risk of multicollinearity. Variables passing this screening were subsequently entered into a multivariable logistic regression to identify independent predictors of HEV seropositivity. Model construction followed a stepwise forward selection procedure guided by the Akaike Information Criterion (AIC), with variables retained in the model if they achieved statistical significance at *p* < 0.05 (Wald test). Final results are presented as adjusted PORs with 95% CIs. Model fit was assessed with the Hosmer–Lemeshow test, and plausible first-order interactions between retained predictors were examined to determine whether combinations of factors significantly modified the risk of HEV infection.

All statistical analyses were performed using Python (version 3.13), employing libraries including pandas, NumPy, SciPy, and statsmodels (18).

## Results

### Herd and sample characteristics

A total of 437 pig blood samples were collected from 87 farms in 7 cantons of the FB&H. This sample represented the major pig-producing regions of FB&H as per the 2023 census. Of the 437 pigs sampled, 315 (72.1%) were growers or fatteners (approximately 3–12 months old), and 122 (27.9%) were adult pigs (>12 months, typically breeders). The sex distribution was roughly even: 241 (55.2%) of the pigs sampled were female and 196 (44.8%) were male. The pig farms in our study were generally small-scale, with the average number of adult pigs per sampled farm was about 5 (mean 5.02), and when including piglets the average herd size was about 11 (mean 11.28). Notably, 37 farms (42.5%) had no adult pigs present (only growers) at the time of sampling, and 61 farms (70.1%) had no piglets on site (e.g., farms engaged only in finishing pigs, without breeding sows or litters at that moment). Based on herd size criteria, the majority of the sampled farms were classified as small farms (*n* = 70 farms, 80.4%), with 10 farms (11.5%) classified as medium and 7 farms (8.1%) as large operations. Pasture-based herding systems were employed by all small-scale farms and by 60% of medium-scale farms, whereas the remaining medium-scale farms, together with all large-scale farms, operated under batch production systems. These figures confirm that pig production in FB&H is dominated by smallholder or family-type farms.

### HEV seroprevalence in pigs

Serological testing revealed a high prevalence of HEV exposure in pigs. Out of 437 pigs, 337 tested positive for anti-HEV antibodies by ELISA. The overall animal-level seroprevalence in the tested population was 77.1% (95% CI: 73.0–81.0%). At the farm level, 83 of the 87 farms had at least one seropositive pig, corresponding to a herd-level seroprevalence of 95.4% (95% CI: 88.9–98.7%). HEV-seropositive pigs were detected in every canton that was included in the study, although the proportion of positive animals varied between cantons (range of animal-level seroprevalence by canton: roughly 60% up to 85%; data not shown in table). All cantons, even those with smaller pig populations, had some degree of HEV presence, indicating widespread distribution of the virus in FB&H swine. It is important to notice that there were no equivocal ELISA results, and no positive samples were retested to validate the initial findings.

**Figure 1:**
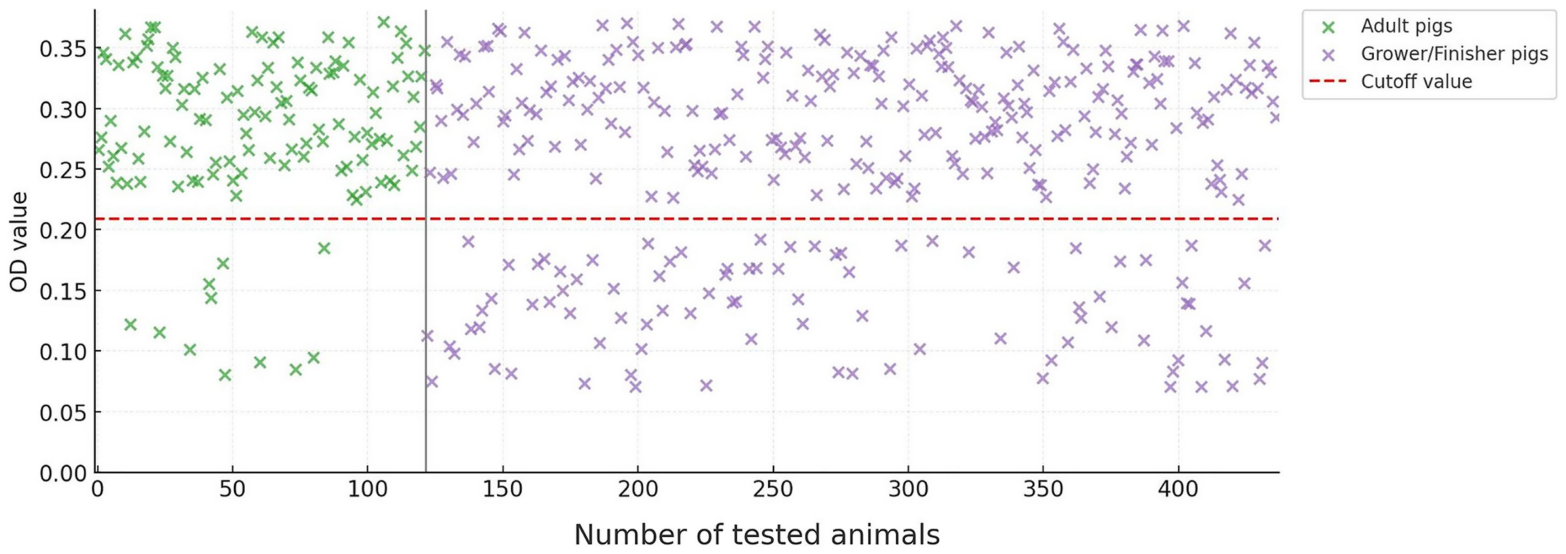
OD_450_ distribution of HEV ELISA readings in adult and grower/finisher pigs in FB&H.

As expected, older pigs were more likely to have HEV antibodies. Among adult pigs, 111 of 122 (91.0%) were seropositive, compared to 226 of 315 (71.7%) grower/finisher pigs (Figure 1). This difference in seropositivity between age groups was statistically significant (*p* < 0.001). There was no significant difference in seroprevalence between male and female pigs.

### Risk factor analysis

Univariable analysis of the questionnaire data identified several farm-level factors associated with HEV seropositivity. Of the 27 variables examined, 7 met the inclusion criterion of *p* < 0.1. These potential risk or protective factors (with their prevalence odds ratios and *p*-values from Chi-square tests) are summarized in Table 1. The distribution of samples across these seven variables, together with their corresponding prevalences, is shown in Table 2.

**Table 1:** Univariable associations of selected farm-level factors with HEV seropositivity in pigs (*N* = 437).

Candidate risk factor	Category	POR	95% CI	*p* (*χ*^2^)
Small farm size	Yes vs. No	20.67	9.03–116.64	0.006
Slatted flooring	Yes vs. No	0.04	0.003–0.49	<0.001
Cleaning frequency	>5 times/month vs. ≤5×/month	0.02	0.001–0.27	<0.001
Wild boars in vicinity of the farm	Yes vs. No	8.87	4.44–77.03	0.088
Swill feeding (feeding food scraps)	Yes vs. No	11.89	5.59–137.78	0.043
Pigs imported in the last 12 months	Yes vs. No	0.04	0.003–0.49	<0.001
Quarantine of newly imported pigs	Yes vs. No	0.10	0.01–0.86	0.028

Eight factors with *p* < 0.1 in Chi-square tests are shown. POR, prevalence odds ratio; CI, confidence interval.

**Table 2:** Distribution of pig samples across the seven potential risk factor variables and their corresponding prevalences.

Variable	Number of tested farms	% (pigs/overall)	Animal-level prevalence (%)
Total	87	100 (437/437)	—
Farm size			
Small	70	27.7 (121/437)	98.3
Medium	10	24.0 (105/437)	81.9
Large	7	48.3 (211/437)	62.6
Floor type			
Straw	62	50.6 (221/437)	91.9
Partially slatted	7	4.6 (20/437)	85.0
Fully slatted	18	44.8 (196/437)	59.7
Frequency of cleaning			
1–2 times per month	11	8.9 (39/437)	100
3–5 times per month	16	20.1 (88/437)	100
>5 times per month	60	71 (317/437)	66.2
Wild boars in the vicinity of the farm			
Yes	47	31.6 (138/437)	99.3
No	40	68.4 (299/437)	66.9
Swill feeding			
Yes	66	50.1 (219/437)	97.3
No	21	49.9 (218/437)	56.9
Introducing new pigs into the herd in the past 12 months			
Yes	73	84.9 (371/437)	80.4
No	14	15.1 (66/437)	100
Quarantine of newly imported pigs			
Yes	67	66.1 (289/437)	73.4
No	20	33.9 (148/437)	84.5

Prior to multivariable model construction, the seven factors were examined for intercorrelations. Two of the factors (‘Slatted flooring’ and ‘Quarantine of newly imported pigs’) were highly collinear, reflecting underlying relationships. The decision of exclusion of variables was based on whichever variable had the stronger univariable association or a more direct interpretability as a risk factor.

Multivariable logistic regression was subsequently performed, including the remaining five candidate factors: farm size (small vs. medium/large), cleaning frequency (>5 times/month vs. ≤5), presence of wild boars in the vicinity of the farm (yes/no), swill feeding (yes/no), and pig importation (yes/no). The last factor (pigs imported in the last 12 months) did not retain significance in the multivariable context (*p* = 0.23) and was dropped, as its inclusion also weakened the overall model fit. The final logistic regression model thus included four predictor variables, all of which were significantly associated with HEV seropositivity (Table 3).

**Table 3:** Final multivariable logistic regression model of independent risk factors for HEV seropositivity in pigs.

Risk factor	Comparison (exposed vs baseline)	Adjusted POR	95% CI	*p* value
Farm size	Small vs. Medium/Large	18.35	11.42–57.9	<0.001
Cleaning frequency	>5 times/month vs. ≤5 times/month	0.01	<0.001–0.15	<0.001
Wild boars in vicinity of the farm	Yes vs. No	3.11	2.13–21.51	0.043
Swill feeding practice	Yes vs. No	5.70	1.14–14.27	0.031

Adjusted POR > 1 indicates higher odds of HEV on farms with the factor; POR < 1 indicates a protective factor.

No significant interactions were found between the main effects in the model (e.g., the combination of small farm and wild boar presence did not deviate from multiplicative expectations).

## Discussion

This study provides the first data on HEV in B&H and assesses the prevalence of HEV in pigs in FB&H. The serological results show that approximately three in every four pigs have been exposed to HEV, and almost all pig farms (95%) have had the virus. Moreover, A farm-level seroprevalence of 95.4% (*n* = 83/87) and an individual-animal seroprevalence of 77.1% (*n* = 337/437) were observed across all cantons, providing strong evidence that *Paslahepevirus balayani* is highly endemic in domestic pigs in FB&H and likely nationwide. These figures are broadly in line with studies from neighboring and other European countries, confirming that B&H’s situation mirrors the regional trend of widespread HEV in swine.

Notably, our observed animal-level seroprevalence of 77% is on the higher end of the range reported in the Balkans, which spans roughly 20–90% depending on the study population and assay (8). For instance, surveys in Serbia and Bulgaria typically found around one-third to one-half of pigs seropositive (8–10), whereas a few studies in Croatia reported farm-level seroprevalences up to 92% (19). The high prevalence likely reflects the inclusion of older breeding animals and numerous small backyard herds, as in endemic settings most pigs have seroconverted to HEV by adulthood. This age-related pattern of seropositivity is consistent with the known epidemiology of HEV on farms: exposure accumulates over time, and older pigs are more likely to have encountered the virus (20, 21). Other studies have identified age as a key predictor of HEV status, with seroprevalence rising sharply in pigs beyond 3–6 months of age (22). Our data support that timeline, as a substantial proportion of fattening pigs (which in FB&H are typically slaughtered at 6–12 months) were already antibody-positive, and nearly all sows and boars (kept beyond 1 year) had been infected at some point.

From a One Health perspective, the finding that HEV circulates extensively in B&H pigs has important public health implications. In Europe and in some extent North and Central America, HEV genotype 3 has transitioned from a travel-related pathogen to a domestically acquired zoonosis, largely due to the swine reservoir (7, 21, 23). Humans can contract HEV through consumption of undercooked pork or liver products or through occupational contact with pigs (2). The high herd prevalence (95%) observed in FB&H means that most farms are potential sources of HEV contamination in the pork production chain. Even if only a fraction of pigs actively shed virus at any given time, the sheer ubiquity of exposure raises the risk that infectious pigs or pig products enter circulation. While the present research did not directly assess HEV viremia or shedding in pigs, other research has shown that in endemic farms a certain percentage of pigs (often 5–30%) may have ongoing infection and virus in blood, feces, or liver at slaughter age (4). Thus, there is a considerable likelihood that HEV has already contaminated, or will contaminate, the food supply in B&H.

The presence of wild boar in the immediate vicinity of domestic pig farms in the FB&H was identified as a significant risk factor for HEV infection in swine (POR = 3.11; *p* < 0.04), corroborating findings from prior studies (24). Molecular analyses conducted in Croatia (25), Sweden (26), and France (27) have demonstrated a high degree of genetic similarity between *Paslahepevirus balayani* isolates from domestic and wild swine, reinforcing the hypothesis of wildlife-livestock transmission. Notably, isolates exhibiting close relatedness to wild-boar strains were predominantly derived from small- to medium-scale farms. In this study, pigs reared on small holdings exhibited a markedly greater predisposition to HEV infection (POR = 18.35; *p* < 0.001) compared to those on larger operations, likely reflecting the lower biosecurity standards characteristic of small-scale farms—a factor also implicated in the 2023 African swine fever epizootic in B&H. Fecal shedding during asymptomatic infection facilitates environmental dissemination via the oro-fecal route, complicating control efforts. Nonetheless, rigorous hygiene measures, including the regular cleaning and disinfection of pig housing, are critical for limiting virus circulation and reducing zoonotic transmission (28). Indeed, infrequent removal of manure from pig pens (i.e., cleaning fewer than five times per month) emerged as one of the most significant risk factors for farm-level HEV occurrence, whereas more frequent cleaning conferred strong protective effects (POR = 0.01; *p* < 0.001).

Feeding swill has also been shown to significantly increase HEV prevalence (29). Evidence from multiple studies indicates that more than half of commercially available pork products have tested positive for HEV (30, 31). Although legally prohibited in FB&H, swill feeding remains widespread among smallholders and was identified here as a risk factor for HEV on swine farms (POR = 5.7; *p* = 0.031). An epidemiological survey in Slovenia further confirmed that all swine-derived *Paslahepevirus balayani* isolates belonged to genotype 3, underscoring the zoonotic potential of HEV across the Balkans and Europe (32). Lapses in farm biosecurity may facilitate viral entry into the environment, particularly via stagnant water sources, which can precipitate human HEV outbreaks (8).

In humans, HEV 3 infections generally last 4–6 weeks and are asymptomatic; however, this genotype is associated with a case fatality rate of up to 10% among symptomatic individuals (6). Understanding the risk factors that sustain HEV persistence in domestic swine is therefore vital for preventing zoonotic spillover. Severe outcomes primarily occur in patients with comorbidities, where iatrogenic transmission through blood transfusion or organ transplantation, common in this group, has been documented (5). High HEV 3 seroprevalences (16–52%) have been reported among blood recipients in the United States, England, Denmark, Switzerland, Germany, and France (33). Occupational or recreational contact with domestic and wild pigs significantly elevates human HEV risk (34). In Bosnia and Herzegovina, routine screening for HEV antibodies in blood donors and transplant recipients is not conducted. Yet, the data from the Institutes of Public Health of B&H for 2023 (35) document 26 cases of human acute viral hepatitis of unknown etiology. Given that hepatitis A, B, and C have been excluded, these figures strongly suggest potentially undiagnosed HEV infections in the human population. Considering the widespread presence of HEV on pig farms across all surveyed cantons in FB&H, and likely in humans nationwide, substandard biosecurity and inadequate zoonotic transmission controls constitute a major public health challenge. An integrated prevention and control strategy, encompassing health services, veterinary authorities, agricultural stakeholders, and the general public, is essential for the development and implementation of effective measures to curb the spread of hepatitis E.

However, several limitations should be acknowledged when interpreting findings of the present study. This research relied exclusively on serological testing without molecular confirmation, which prevents assessment of active infection or viral genotypes. The cross-sectional design provides only a single time-point snapshot, so temporal patterns of HEV circulation could not be evaluated. Information on management and biosecurity practices was partly derived from self-reported data, which may introduce reporting bias, and several predictors yielded wide confidence intervals, warranting cautious interpretation of these results. Finally, the absence of molecular and spatial data limits the depth of epidemiological interpretation.

## Data Availability

The raw, anonymized data supporting the conclusions of this article will be made available by the authors, without undue reservation.
